# Wellbeing for young elite musicians: development of a health protocol from a student perspective

**DOI:** 10.3389/fpsyg.2025.1401511

**Published:** 2025-02-12

**Authors:** Ann Shoebridge, Margaret S. Osborne

**Affiliations:** ^1^Melbourne Conservatorium of Music, The University of Melbourne, Parkville, VIC, Australia; ^2^Melbourne School of Psychological Sciences, The University of Melbourne, Parkville, VIC, Australia

**Keywords:** health promotion, performance-related problems, music student, musician, conservatoire, tertiary music, wellbeing, qualitative

## Abstract

Musicians’ vulnerability to psychological and physical problems provides a compelling argument to include health and wellbeing training in music education. This study forms part of a larger project to design an evidence-based wellbeing protocol for young elite musicians at a pre-professional music training institute. Recommendations from the health, education, performance science, behavior change, and occupational and public health literature provided theoretical and practical foundations for the project. The aim of this study was to identify barriers to wellbeing and strengths of an existing health protocol, and provide recommendations for change from the student perspective. Four in-person focus groups were held on the same day, attended by 68% (*n* = 45) of the student cohort. Semi-structured discussions were recorded, transcribed and analyzed thematically. Barriers to wellbeing and recommendations for change were collated from the data and distributed to the student body to be rated in priority order. Participants appreciated the existing program for its holistic approach delivered by a range of skilled practitioners. Barriers to wellbeing included constraints in finances, leave allowances and time, pressure from interpersonal challenges and unhealthy norms in the music performance culture. Recommendations for change included updating the financial policy; having more flexible leave conditions to allow higher earnings and better access to performance opportunities; having reliably scheduled time off; collaborative planning with staff around playing rosters; more activities for musician enhancement and social bonding; exercise opportunities in-house; addressing pervasive cultural norms around practice and breaks; education regarding respectful and professional conduct; an effective complaints procedure, and more practical wellbeing sessions.

## Introduction

Musicians in pursuit of excellence must develop exceptional psychological and physical skills in environments that often present significant challenges to mental and physical health. Mental health studies have reported over 75% of professional musicians and around 70% of tertiary music students have at some time experienced anxiety or depression (Gross and Musgrave, 2016; Koops and Kuebel, 2019). A systematic review of prevalence and risk factors for musculoskeletal pain in musicians reported lifetime prevalence ranging between 46 and 90%, and point prevalence ranging between 9 and 60% depending on study methodology (Rodríguez-Gude et al., 2022). Symptoms arising from playing an instrument or singing can appear as early as childhood, with higher prevalence estimates reported in musicians with more playing experience (Ackermann et al., 2012; Cruder et al., 2020; Ranelli et al., 2015). The extent of playing-related problems and investigation into contributing risk factors has received considerable attention, with music and health experts recommending multifaceted approaches to musicians’ health promotion and education (Araújo and Spahn, 2022; Chesky et al., 2006; Ginsborg et al., 2012; Matei and Phillips, 2023). The expansion of work in this area has raised awareness of playing-related problems and led to interdisciplinary health initiatives tailored for musicians, including Healthy Conservatoires UK, the NASM requirements for music schools in the US, and specialist musicians’ clinics in Germany (Healthy Conservatoires, 2018; NASM 2019-2020, n.d.; Rosset et al., 2022; Zalpour et al., 2021). However, studies on health programs with musicians have delivered mixed results (Rotter et al., 2020; Stanhope et al., 2020), and there is no gold standard approach to preventing playing-related problems. The success of public health and occupational health initiatives in improving population health and wellbeing and reducing workplace injury suggests that these and allied disciplines may offer perspectives or processes that could be useful for improving the health status of musicians. This article presents a discussion of multidisciplinary frameworks and interventions that informed development of a health protocol design for young elite musicians, and enlarges on the stakeholder engagement phase of the protocol development to present student perspectives on wellbeing and recommendations to help them to thrive.

### The problem

Musicians experience more psychological and physical problems than the general population (Loveday et al., 2022; Vaag et al., 2016), while also delivering higher scores in positive psychology and emotional regulation (Ascenso et al., 2018; Athalia and Kilis, 2020). In a pre-COVID study with over 2,000 UK professional musicians, 71% reported anxiety or panic attacks, and 69% reported depression resulting from precarity of employment opportunities and tenure, income uncertainty, and challenges related to damaging industry cultural norms and working conditions (Gross and Musgrave, 2016). In terms of physical health, up to 90% of professional and 89% of tertiary musicians report physical symptoms related to music performance (Ackermann et al., 2012; Ioannou et al., 2018; Steinmetz et al., 2015). These statistics represent unacceptably poor levels of occupational health among musicians that are accompanied by substantial personal and social costs.

Ideally, healthy performance principles would be integral to music education from the outset and underpin a professional musician’s work (Rosset et al., 2022). As the primary contact and source of advice for students experiencing music-related health issues, music teachers are concerned about students experiencing music-related health issues (Norton, 2016). However, other than in countries such as Austria, Germany or Switzerland where music physiology training for music teachers is well-established, teachers’ knowledge of wellbeing and playing-related problems may be guided more by personal experiences of symptoms and symptom management rather than on a working knowledge of the complex factors that contribute to playing-related problems (Norton, 2016). Consideration of wellbeing and playing-related problems is not generally included in elementary music instruction, at least partly due to teachers’ limited knowledge of playing-related problems and due also to the focus being on acquiring music skills and on performance rather than the musician’s condition.

Higher education offers a structured environment where students’ playing health can be influenced positively while they are still in an active learning context (Rosset et al., 2022). Student wellbeing programs are more common in universities and conservatoires now than they were in the past, but many music faculties are yet to include wellbeing initiatives that cater for the specific needs of musicians (Wijsman and Ackermann, 2019). Where they are present, health initiatives offered by tertiary music faculties typically comprise information and advice, optional involvement with wellness activities, symptom treatment through student services, and perhaps musicians’ health as an elective rather than a core subject. With few exceptions, musicians’ health education programs provide information but do not actively facilitate behavior change, even though information on its own is not sufficient to change behavior and does not necessarily improve health status (Kelly and Barker, 2016; Michie et al., 2011; Rosset et al., 2022). Additional influences on music students’ health are the conservatoire environment itself, and that music students appear to exercise low responsibility for self-care (Araújo et al., 2017; Perkins et al., 2017).

While the higher education setting is well-placed to improve musicians’ health in the long term, the overarching work environment is likely to exert a stronger influence on health and health behavior than individual knowledge or skills (Oakman et al., 2018; Weale et al., 2022). Psychosocial as well as biomechanical hazards at an organizational level have a higher impact on psychological and physical health than education or protective equipment for individuals in the work environment (NIOSH, 2016; Oakman et al., 2018). However, it is unclear whether this is known or how much it is acted upon in professional or educational music settings, where for the most part health initiatives are implemented only at the musician level rather than also working with organizational structures and expectations. Our research questions for designing a wellbeing protocol for young elite musicians were:

1. What are the structures, processes and content that influence musicians’ wellbeing?
2. What needs to be done to reduce musicians’ health problems?

A review of the interdisciplinary literature was the first stage in answering these two questions.

### Wellbeing definition

The need to thrive and not just survive is expressed in the World Health Organisation’s (WHO) definition of health as “a state of complete physical, mental and social well-being and not merely the absence of disease or infirmity” (WHO, 2020, p.1), and health also as “a resource for everyday life, not the objective of living. Health is a positive concept emphasizing social and personal resources, as well as physical capacities” (WHO, 1986, p. iii). The WHO’s biopsychosocial concept of wellbeing encompasses the spectrum of health promotion, problem prevention, early intervention, and treatment of psychological and physical conditions in a given socio-cultural context. Therefore, the terms “health” and “wellbeing” are used interchangeably in this article.

**Figure 1:**
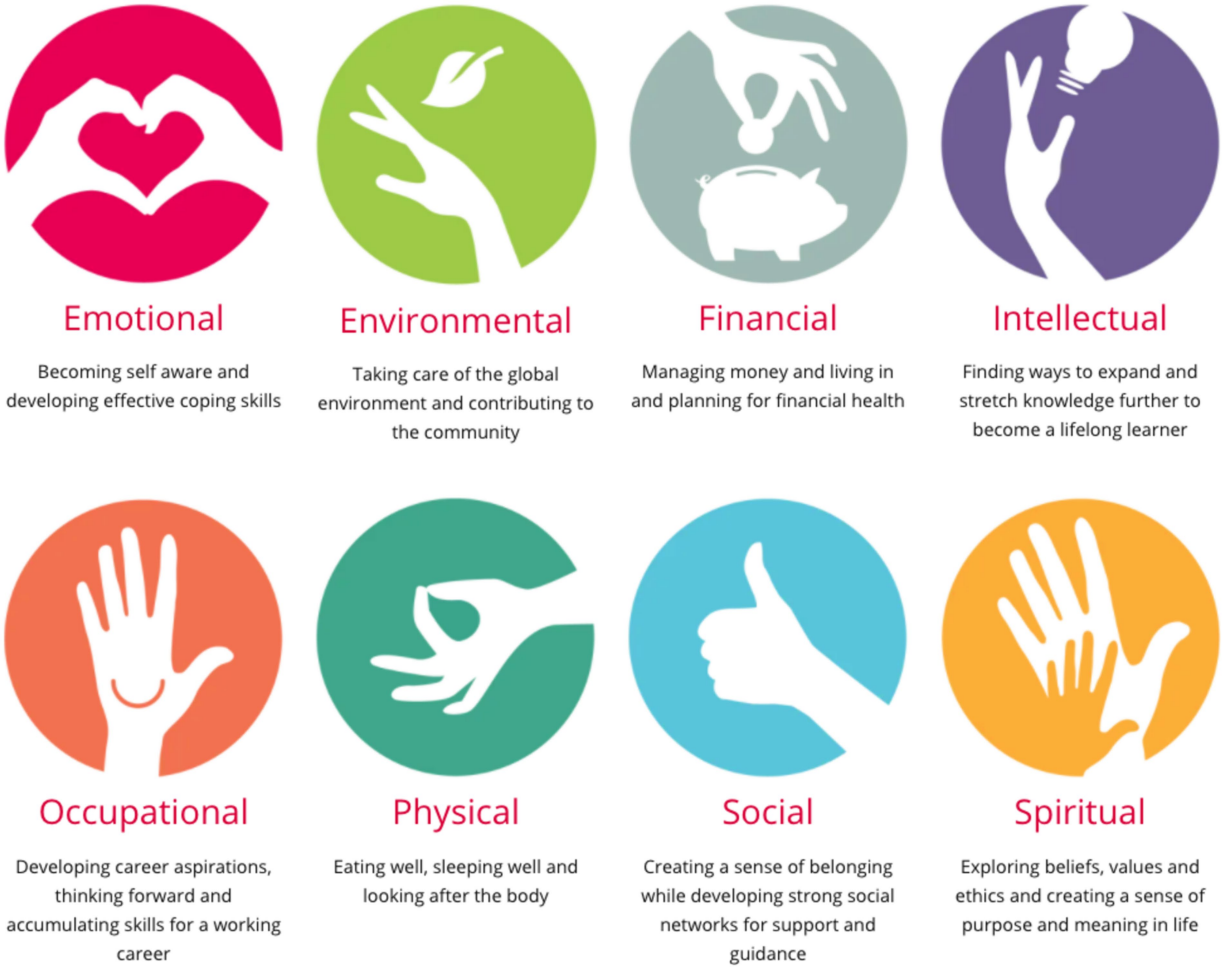
Healthy conservatoires wellbeing framework. Reproduced from https://healthyconservatoires.org/framework/. Used with permission.

In keeping with the WHO’s definition of wellbeing, the Healthy Conservatoires (2020) Fit to Perform eight-factor framework captures the multidimensional nature of performer wellbeing (Figure 1). A healthy university is one that: “aspires to create a learning environment and organizational culture that enhances the health, wellbeing and sustainability of its community and enables people to achieve their full potential” (Dooris et al., 2010, p. i). Music performance demands high physical and emotional engagement with appropriate levels of fitness to meet those demands. It is not enough to be injury-free; musicians need to have the resilience to perform at their best under considerable pressure (Araújo et al., 2017).

A unifying aspect of the WHO and Healthy Conservatoires’ wellbeing models is that wellness is multidimensional. Wellbeing research represents psychological health as positive emotion combined with the practice of self-care, making meaning, and having a sense of personal agency within the social environment (Keyes, 2009; Ryff and Singer, 2008). Keyes’ (2009) assessment of mental health ranging from robust mental health (flourishing), through to poor mental health (languishing), provides a useful tool for mapping change in response to wellbeing initiatives. Self-Determination Theory specifies three core psychological needs that determine wellness: *competence* (the desire to feel effective); *relatedness* (the need to feel connected to others), and *autonomy* (sense of choice or personal control). Growth and psychological wellbeing result when these core needs are fulfilled; conversely, psychological illbeing arises when these needs are not met (Evans, 2015).

### Interdisciplinary perspectives

In view of the ongoing prevalence of playing-related problems despite several decades of research into musicians’ health, we expanded on emerging directions in performance science (Araújo et al., 2017; Gross and Musgrave, 2016; Perkins et al., 2017), to source perspectives from disciplines that have invested significant resources over time in reducing costs associated with injury and ill-health, including public and occupational health. In keeping with the recognition that health literacy requires behavior change as well as education (Kelly and Barker, 2016; Nutbeam, 2019), additional perspectives were sought from the behavior change and health promotion literature. This shifts the focus of musicians’ health, usually centered on biomedical constructs, to recognizing contextual and choice elements in addressing the problem of playing-related problems.

#### Behavior change

Behavior change involves working with behavioral determinants to bring about constructive change (Michie et al., 2008). There is a growing realization that if health outcomes are to improve in the music environment, education must move beyond providing information to changing behavior (Ajzen et al., 2011; Araújo and Spahn, 2022; Matei and Ginsborg, 2022). Ajzen et al.’s (2011) Theory of Planned Behavior proposes that behavior arises from a complex mixture of attitudes, subjective norms and perceptions of behavioral control which are affected by context but not reliably by information, while Araújo and Spahn (2022) concluded that as well as appropriate information, healthy behaviors in musicians require healthy musician-centered settings that foster healthy decision-making and practices. Matei and Ginsborg (2022) reported positive engagement with health and increased adoption of healthy behaviors in some participants following a 5-month health module. However, other participants identified a number of barriers to engaging practically with the course material, some of which were that they felt the program did not apply specifically to their situation, or was not sufficiently practical or solution-focused, or was partially redundant.

**Figure 2:**
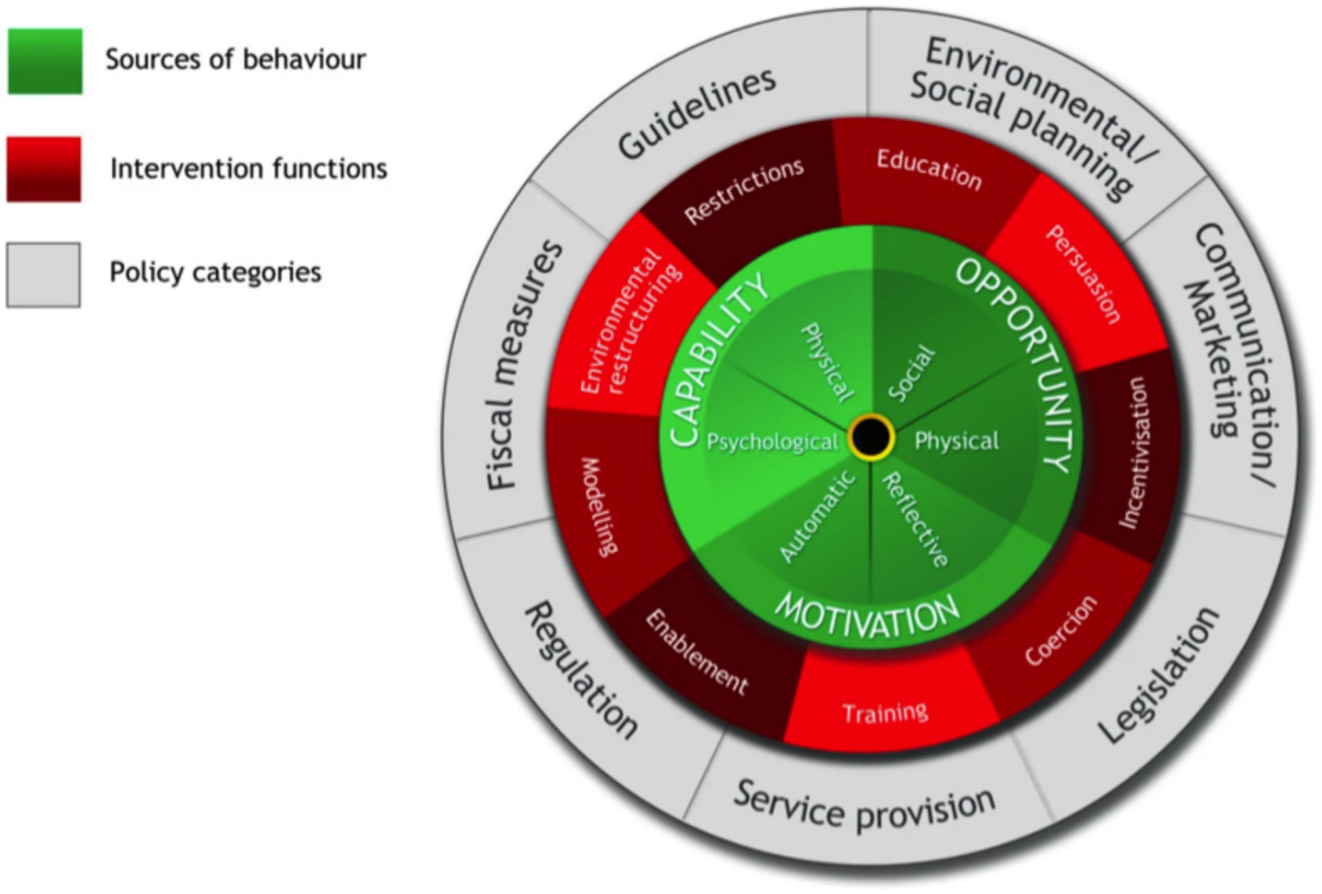
COM-B: The Behaviour Change Wheel. Reproduced from Michie et al. (2011, p. 7). Used with permission.

According to Michie et al. (2011), behavior change is characterized by three main drivers: Capability, Opportunity and Motivation. The COM-B model of behavior change elaborates on these drivers to provide a framework to achieve positive changes in individual health behavior within a psychosocial environment applicable across individual, institutional, and legislative domains (Figure 2). Resistance to change at one level can affect factors lower in the hierarchy, effectively locking in a chain of systemic attitudes or behaviors, positively or negatively. The COM-B model moves beyond the customary focus on knowledge and behaviors of individual musicians to include the institutional and social layers that actively influence behavior. Musicians’ health has previously been considered through the lens of the COM-B model (Norton, 2020) and used to effect changes in health behavior as part of the Music Impact study (Matei and Ginsborg, 2022).

#### Occupational and public health

A review of international guidelines for workplace mental health scored Canada’s *Psychological Health and Safety in the Workplace* (CSA Group and BNQ, 2013/2018) highest of all reviewed guidelines for well-researched, rigorous design that included comprehensive guidance and implementation tools at every level of an integrated approach to mental health (Memish et al., 2017). Canada’s guidelines specify three “strategic pillars” for psychological health and safety: prevention of harm, promotion of health, and resolution of incidents or concerns. These strategic pillars parallel levels 3, 4 and 5 of the WHO’s 10 essential public health operations: health protection, health promotion, and disease prevention/ management, including early detection of illness (WHO, 2021).

**Figure 3:**
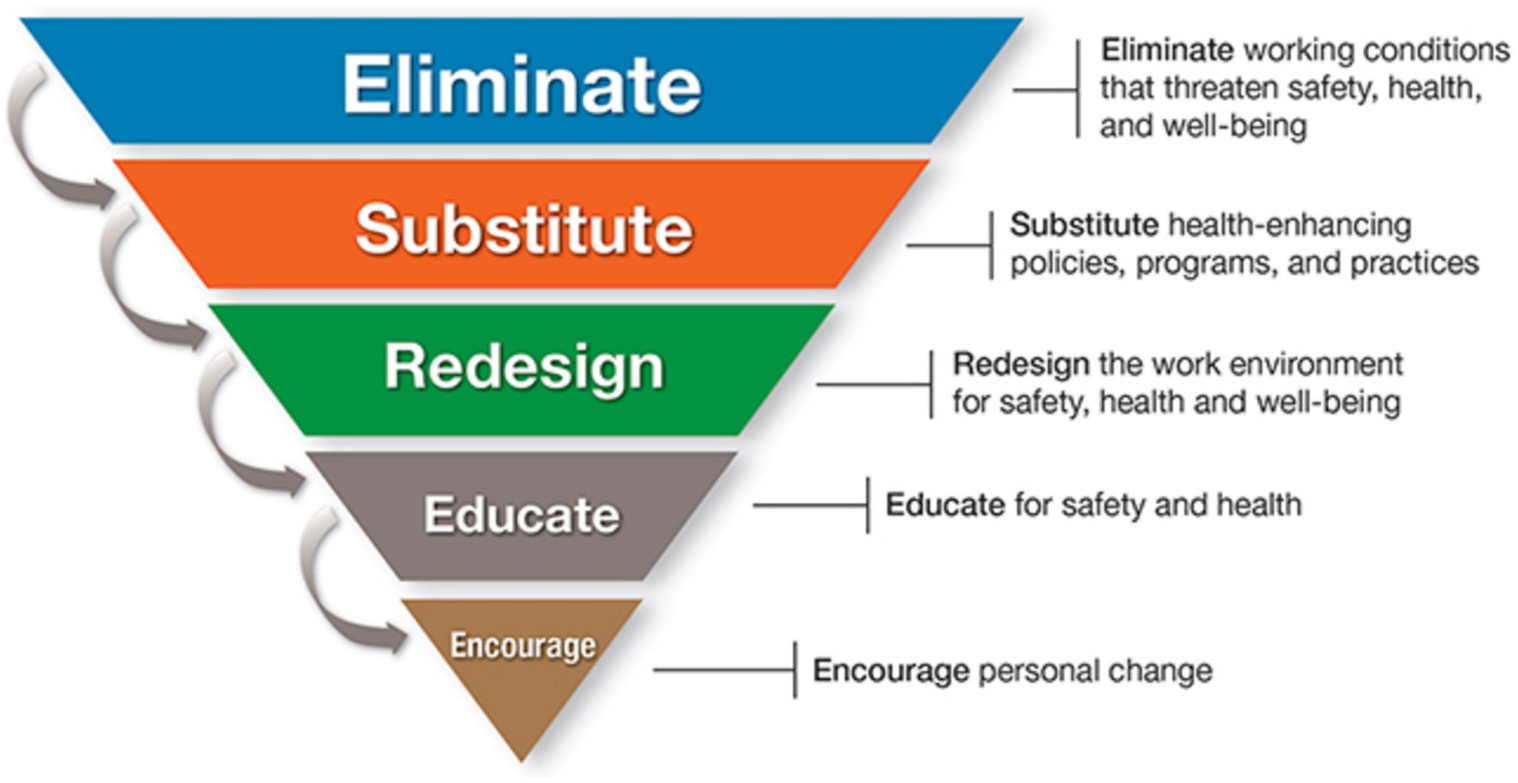
Hierarchy of Controls Applied to NIOSH (National Institute for Occupational Safety and Health) *Total Worker Health* ®. Reproduced from NIOSH (2016).

Working conditions including policies, practices, programs and the work environment have been identified in the hierarchy of control as the most influential factors in workers’ health (Figure 3) (NIOSH, 2016). When institutions actively create safe work environments through policy and practical strategies in the work environment, they are operating at the highest possible level of health and safety. Although positive change needs to be shown to happen at the individual level, targeting individual behaviors has the least traction in the health and safety hierarchy.

Recent performance science research parallels recommendations in occupational health to understand not just ill health, but wellness in the work context, and to attend to the institutional and social culture within which musicians work (Araújo and Spahn, 2022; NIOSH, 2016; Oakman et al., 2018; Perkins et al., 2017). It is the role of institutions to advocate for and support healthy cultures and healthy work practices based on contextual risks.

A key factor in Canada’s approach to workplace psychological health is active participation of stakeholders in identifying health and safety issues and engaging in all aspects of program development, implementation and evaluation. Worker consultation enables clearer identification of barriers to health and improves the effectiveness and sustainability of health initiatives (NIOSH, 2016). The NIOSH guidelines stress that an organization’s health protocol is unlikely to achieve significant success unless gaps in psychological safety are assessed and needs are addressed before embarking on active health promotion (CSA Group and BNQ, 2013/2018). A combined top-down and bottom-up approach is held to be more effective than the top-down approach usually taken. Wellbeing initiatives must be designed and tested in context if they are to be effective (Weale et al., 2022). Stakeholder consultation and program review are especially critical in view of the point that there is no one-size-fits-all approach to wellbeing.

Evidence-based interventions are considered to be the gold standard for health initiatives. However, even where evidence of intervention effectiveness exists, an acknowledged gap separates research evidence and its translation into practice (Gentry et al., 2020; Verhagen et al., 2014). Linear models of health policy, where scientific findings are directly applied to real-life contexts, can fall short of meeting complex “real-world” situations (Gentry et al., 2020). Although numbers derived from linear research methods provide a valuable picture of the extent of playing-related problems and any changes to that picture in response to intervention, they do not capture the complexities that drive health behavior, including the dynamic psychosocial environment that influences minute-to-minute behavior choices. Epidemiologists recommend bridging the research-practice gap by including representative stakeholders in reviewing evidence, devising effective practices or products for their population, and ensuring the success of these practices by evaluation and adjustment (Verhagen et al., 2014). Verhagen et al. (2014) recommend evaluating the impact of any intervention on the population at both individual and organizational levels.

#### Health education and health promotion

Education aims to provide access to knowledge and skills, training in practices and traditions, to equip students with tools to be life-long learners and to responsibly exercise their full potential (Biesta, 2020). There has been a shift in education over the last two decades from traditional top-down approaches focused on inputs to more active student engagement in the learning process (Biesta, 2020). Contemporary educational theory argues that higher education should be transformative, not merely involve transmission of information and skill acquisition (Paul and Quiggin, 2020). In line with educational theory, health education needs to move beyond learning about healthy behaviors and aim to change beliefs, attitudes, and the choices people make (Nutbeam, 2019).

Health literacy as a goal of health promotion is seen as building skills and abilities across the lifespan to adopt healthy behaviors in the face of complex changing circumstances. Context is important to the exercise of health literacy; people with high levels of health literacy can make unhealthy decisions under the pressure of environmental influences that make it difficult to choose in favor of one’s own wellbeing (Kelly and Barker, 2016). The teaching of personal skills and strategies must be supported by attention to the social, economic and cultural environment (Nutbeam, 2019). The shift away from the top-down model in educational theory echoes the shift from early concepts of health education centered on personal risk exposure and lifestyle choices to the WHO’s adoption of a health promotion paradigm that includes five strategies: “build healthy public policy, create supportive environments for health, strengthen community actions, develop personal skills and reorient health services.” (Nutbeam, 2019, p. 706).

### Health protocol design

#### Consultation and engagement

The success of a workplace health protocol depends on stakeholder engagement at every stage from the design phase onward (NIOSH, 2016). Competing interests between stakeholders including health professionals, administrators, researchers, workers and workers’ families can prevent effective realization of evidence-based health policy (Cherniack and Lahiri, 2010). Additional obstacles to effecting positive changes in workplace health include health practitioners or others offering advice on the basis of cultural values or beliefs rather than on evidence, and service provision based on health care interests rather than appropriately fitting workers’ needs (Cherniack and Lahiri, 2010). A further complication can arise for musicians trying to recover from injury who receive conflicting advice from health practitioners. Musicians already having difficulty accessing appropriate treatment, trying to make sense of the array of options on offer, and trying to balance injury and a playing schedule can be confused by having to navigate contradictory advice from different professionals (Price and Watson, 2011). This highlights the need for a cohesive team approach, for an understanding of best practice among support professionals, and for a central objective serving musicians’ performance goals. The whole-system settings-based approach espoused by Healthy Conservatoires Network UK reflects this principle (Araújo et al., 2017).

#### Ongoing program evaluation and adjustment

The success of health promotion in occupational settings hinges on continuous evaluation and adjustment (CSA Group and BNQ, 2013/2018). However, few ongoing programs have been assessed for their impact on reducing playing-related pain, with none known to the authors undergoing regular ongoing evaluation.

A scoping review of health education programs for music students and teachers suggested health education with injury prevention strategies reduced playing-related pain and music performance anxiety (Evans et al., 2024). However, as scoping reviews are designed to capture key concepts rather than evaluate the quality of the evidence, the reliability of results from the included studies is unclear. Systematic reviews of intervention studies with musicians have reported inconsistent results with respect to improving participants’ health (Rotter et al., 2020; Stanhope et al., 2020). One systematic review of 20 intervention studies indicated that incorporating strength training in preventative programs might reduce musculoskeletal complaints among musicians in the short term (Laseur et al., 2023). Matei and Ginsborg (2022) found that a health education course increased knowledge and self-efficacy among first-year music students but did not significantly improve health behaviors or reduce PRMD frequency and severity. Similarly, Rosset et al. (2022) reported increased knowledge and skills from a compulsory health education program with first year students, but no significant changes in health attitudes, pain, impairments, general health, mental health, or performance anxiety. A 2-year review of a musician-specific preventative health curriculum given to first year university students by Zander and colleagues (Zander et al., 2010) found that psychological health and performance skills stabilized, with no reduction in physical symptoms. Some evaluated programs show improvements; however, study quality varies, and long-term evidence is lacking (Laseur et al., 2023). To address limitations, these studies recommend understanding the needs of music students as essential for developing effective courses and support measures from the onset of education. Consulting best-practice literature and employing iterative processes with rigorous investigations, including pilot studies and a range of validated measures, is crucial. Methodological improvements such as larger sample sizes, stratification by instrument sub-group to enhance targeted interventions and assist in meta-analyses, and longer follow-up periods are recommended. Interventions at both the institutional and individual levels should be evaluated for their timing, duration, and impact on disease processes (Verhagen et al., 2014).

Interdisciplinary recommendations to achieve a successful health protocol are summarized in Table 1.

**Table 1:** Interdisciplinary recommendations for implementing a successful health protocol.

	Recommendation	Source
1	Use appropriate theoretical frameworks through all stages of research, planning, intervention and evaluation of wellbeing initiatives.	Willis et al. (2019)
2	Implement multi-tiered strategies across organizational culture and governance as well as individual interventions.	CSA Group and BNQ (2013/2018), Lowe (2004), Matei and Ginsborg (2022), Michie et al. (2011), Nutbeam (2019), Oakman et al. (2018), Perkins et al. (2017), and Sorenson et al. (2013)
3	Research work instead of focusing on problems as the main source of information.	Rae et al. (2020)
4	Identify systemic, contextual, personal, physical and psychosocial risk factors and the relationships between them using quantitative and qualitative research methods.	Macdonald and Oakman (2015), and Sorenson et al. (2013)
5	Consult stakeholders throughout the health initiative, including researching, planning, implementation, ongoing program evaluation and adjustment.	CSA Group and BNQ (2013/2018), Lowe (2004), Sorenson et al. (2013), and Verhagen et al. (2014)
6	Address attitudes and processes within the institution that do not align with wellbeing goals.	CSA Group and BNQ (2013/2018), Matei and Ginsborg (2022), Nutbeam (2019), and Perkins et al. (2017)
7	Change behavior rather than just improve health knowledge	Kelly and Barker (2016), Matei and Ginsborg (2022), and Michie et al. (2011)
8	Address risk factors at highest level of control hierarchy.	Oakman et al. (2018)
9	Consider the legislative and social environment in which the institution is situated.	Kelly and Barker (2016), Michie et al. (2011), and Nutbeam (2019)
10	Ensure health initiatives are appropriate and relevant to the context.	Lowe (2004) and Nutbeam (2019)
11	Everyone being responsible for workplace health; managers assume health advocacy roles.	CSA Group and BNQ (2013/2018), Lowe (2004), and Sorenson et al. (2013)
12	Evaluate whether clearly defined wellbeing goals are being met and adjust program accordingly on an ongoing basis.	CSA Group and BNQ (2013/2018), Lowe (2004), Matei et al. (2018), Nutbeam (2019), Sorenson et al. (2013), and Verhagen et al. (2014)

### Study context

The brief for the overall project was to design and implement a benchmark wellbeing protocol for a pre-professional training institute that is sustainable, reduces occupational, psychological, and physical problems, and enhances musicians’ overall wellbeing.

**Figure 4:**
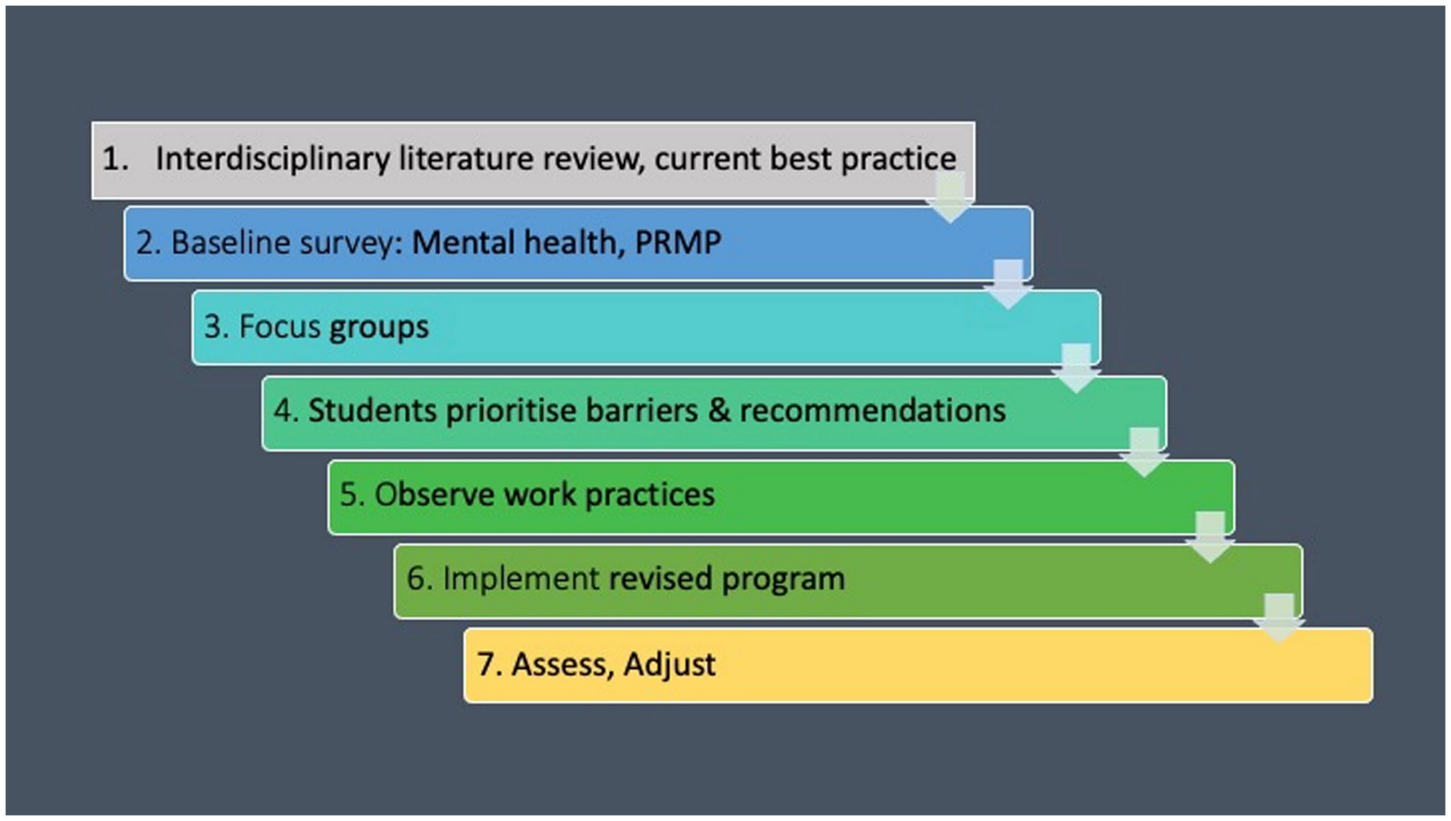
Protocol design 7-step process.

We designed a 7-step theoretically-driven process based on recommendations from the occupational and public health literature, and from wellbeing and behavior change theories (Figure 4). The first step was to conduct the literature review as described above to provide an evidence base for best practice in implementing a health protocol for musicians. The second step was to conduct a quantitative online health and wellbeing survey to map students’ psychological and physical health status. For the third and fourth steps of the project reported here, we conducted focus groups with student participants to gain a contextualized understanding of the survey results, and from the focus group results we synthesized barriers to wellbeing and recommendations for change to guide improvements to the institute’s existing wellbeing protocol. The online survey and focus groups were designed to provide baseline data to assess wellbeing protocol effectiveness and inform responsive change. Step 5 was to observe work practices *in situ* to identify any unconscious agendas or work practices that might not have been mentioned during the focus groups, but that may be affecting wellbeing. Step 6 was to implement a revised program based on student health data and recommendations for change, and best practice from the literature. Step 7 is recommended as a repeating ongoing exercise to ensure continuing relevance and effectiveness of the wellbeing protocol.

### Aim

The aim of this study, which includes the third and fourth steps of the overall project, was to identify strengths in the existing wellbeing supports at a pre-professional training institute for elite classical musicians from the student perspective, and synthesize student views on barriers to wellness and recommendations for improvements to the existing health protocol.

## Materials and methods

A qualitative approach using a social constructivist framework was used for this study. The social constructivist framework rests on the principle that knowledge is not a fixed entity, but constructed from the meaning individuals give to their experiences within their social environment, which are subjectively interpreted within cultural, social and psychological contexts (Pilarska, 2021). The constructivist framework, used in previous education and conservatoire research (Jin et al., 2020; Perkins et al., 2017) recognizes that our backgrounds and experience as musicians’ health researchers, psychological and physical health care professionals and health and performance educators, form our understanding of wellbeing and health behavior. At the same time, constructivism allows the potential for unforeseen and contrasting attitudes and experiences to be introduced by study participants.

In keeping with recommendations from the literature and the complexity of factors that influence wellbeing, we sought the experiences and views of participants as service recipients and key stakeholders in the wellbeing program. All students were advised about the study by the institute’s manager of people and culture at the start of the academic year. The second author (MO) emailed every student with information about the study and an invitation to participate. Participants returned signed consent forms by email.

The study was conducted in two stages. Stage 1 involved running the focus groups and conducting a thematic analysis. Stage 2 involved students ranking the importance of themes as barriers and recommendations.

### Stage 1: Focus groups

According to Kristiansen and Grønkjær (2018, p.1), “interactions between focus group participants can reveal and highlight the participants’ perceptions, attitudes, thinking, and framework of understanding, as well as identifying group norms, subcultural, and cultural values.” As the music culture and the formal learning environment are both cultural entities that influence individual and group experiences, focus groups were used as an appropriate forum to capture participants’ attitudes and responses, with group discussions guided by a semi-structured questionnaire (Supplementary material 1).

Focus groups were scheduled at a time when most students were on campus. To accommodate the number of participants while limiting group size, four focus groups were run on the same afternoon in November 2022, two consecutive groups by each author. Each group ran for between 45 min and an hour. Focus group discussions were recorded with the consent of all participants and transcribed for thematic analysis. The identities of students who attended focus groups remained confidential, known only to the authors and the other members of their focus group.

Thematic analysis using inductive and deductive processes enabled participant responses and attitudes to wellbeing and the wellbeing program, to be distilled and synthesized in the form of acknowledged barriers to wellbeing and recommendations for change for the institute’s use (Braun and Clarke, 2006). Initial coding included gerunds reflecting participants’ actions, and descriptive words and phrases. The final themes resulted from a reflexive process of returning to the transcripts and audio recordings, grouping the codes, and identifying recurring patterns through the narratives (Braun and Clarke, 2013). The thematic analysis provided a structure that moved beyond a summary of student responses to highlighting systemic issues that related back to wellbeing and behavior change theories. In addition to this, specific recommendations for change offered by participants were highlighted in the transcripts and included in the final lists distributed to the students for ranking.

### Stage 2: Ranking of barriers to wellbeing and recommendations for change

Hard copy lists of possible barriers to wellbeing (Supplementary material 2) and recommendations for change (Supplementary material 3) derived from focus group discussions were distributed to students by researchers at a lunchtime gathering in April 2023. Using a participatory method (Vaughn and Jacquez, 2020), students were invited to rank their five most important items on each list in order from 1 to 5. The sheets were completed and returned to the authors at the time by students who chose to do so. The ranked lists were then analyzed to establish which items carried the most weight, and a report with recommendations from the analysis was submitted to the institute’s administration.

## Results

### Participants

Forty-five musicians (response rate 68%) at a prestigious pre-professional music training institute participated in one of four focus groups. Each of the four focus groups included 9–14 participants. The average age of participants was 23 years (SD = 2.09). Twice as many males as females participated in the focus groups, which reflected the proportion enrolled across the program. Further details on age and gender have been withheld to protect the identity of participants.

Entry to the institute is gained through a two-stage audition process. All students play orchestral instruments. Students attend for between 1 and 3 years of individual and ensemble classical music tuition and performance programs which aim to prepare them for life as professional musicians. Applicants must be no older than 28 when they enrol, and most students who enter the institute have completed year 12 of secondary school. Each student is provided with a limited stipend through philanthropic funding, and sources the balance of living expenses through family support or external employment.

### Pre-intervention wellbeing supports

Health supports have been part of musician training at the institute for all year levels from the early 2000’s, instigated by members of the managerial team and teaching staff. Advice was sought from performing arts medicine organizations and health care professionals with expertise in musicians’ health to inform program content and delivery. The institute has developed relationships with trusted external experts who visit to deliver the health components of the program. The program varies somewhat from year to year, but for a little over a decade has included compulsory lectures, workshops, and the opportunity to have individual assessments by a musicians’ audiologist and a physiotherapist. Three free psychology sessions are available for students, as are subsidised custom-made earplugs.

The program provides health and wellbeing education, information on policies around appropriate behavior, and crisis support contacts. Support persons among the institute’s professional staff are clearly nominated for students to approach if experiencing health and wellbeing issues.

The wellbeing program team includes teachers, senior management and administrative faculty, and performing arts health professionals who are experienced in consulting, research and teaching in a high-performance context. External professionals include a physiotherapist, audiologist and psychologists. Topic areas covered in the program include musculoskeletal health, psychological health, body/mind techniques, social/spiritual/environmental matters, and career skills. Social events were scheduled to enhance student wellbeing and facilitate group cohesion. Supplementary material 4 shows specific issues covered under each wellbeing topic area.

Wellbeing sessions have to fit around a packed performance schedule and the availability of external consultants, so they are often spaced at irregular intervals as time and health experts’ schedules permit. This hinders information being presented in a cohesive, scaffolded manner that systematically reinforces and builds on prior learning. This study was the first instance of the wellbeing program being assessed for efficacy.

### Strengths of the existing wellbeing protocol

Participants had a prior understanding that health was both physical and psychological. They understood that being fit to play, sufficient rest, healthy diet, social connection and injury prevention are all part of wellbeing. They spoke about wellbeing as individually experienced, and about the challenge of finding balance in the intense environment of the institute. They noted that when wellbeing suffers, playing is adversely affected.

Participants expressed appreciation for the supportive culture of the institute, the opportunities it created for them as musicians, its commitment to their wellbeing, and for the initiatives offered to support their playing longevity. They felt well-informed about what they should be doing in terms of self-care. Most had not experienced the holistic attitude to performance wellbeing cultivated by the institute prior to entering the program. Elements of the existing wellbeing offerings that participants found helpful are summarized in Table 2.

**Table 2:** Elements of the wellbeing program participants identified as being helpful.

1	The priority placed on wellbeing, with provision of a range of different services and information to meet different needs
2	Health education, resources and services provided by people who are highly skilled in their fields
3	Musicians’ health research presented in an accessible and practical way
4	Learning and performance opportunities that develop skills in how to monitor thinking and attitudes and connect one’s mindset with playing outcomes
5	Physiotherapy expertise in musicians’ health and performance
6	Audiology advice and care
7	Free counselling sessions with choice of psychologists
8	Dietician
9	Having a clear go-to person for any difficulties they were experiencing
10	Group and individual social events including Baroque dancing, staff concerts, and gatherings around food building a sense of community and morale amidst what is otherwise a very demanding life
11	Having a voice in the affairs of the institute through the Musicians’ Committee

### Barriers to wellness and recommendations for change

Barriers to wellness and recommendations for change divided into four main themes, all of which related to the institute’s organizational systems:

1. Finances
2. Leave
3. Time
4. Pressure

Further issues participants raised concerned program structure and content, health and fitness, and their social lives. See Supplementary material 2 for the complete list of barriers to wellness and Supplementary material 3 for the complete list of recommendations for change.

#### Finances

Financial difficulties were experienced as a major stressor. The limited stipend combined with a cap on allowable earnings created pressure to work to earn enough to live on, while simultaneously restricting participants to an income below the amount needed to pay basic living expenses. The conflict between limited opportunities to cover living expenses and practice and playing commitments placed affordable adequate nutrition, time to exercise, rest, and maintaining social connections largely out of reach.

*You cannot just be using the institute’s financial assistance. That’s not going to be enough, so you need to be making work some other way, and so if that works, what you are doing happens to also be coming at the same time as a really busy program, and those can be some exhausting weeks.*

*Group D Speaker 5*

*…and then I spend it all on the food because I’m too knackered to cook… so you are spending the money, it takes energy to work, and then you get the money and you are spending the money on food, literally to live and survive. And that’s exactly, like, I’m not thriving. I’m surviving at that at that point, and then I cannot go home to see my family over summer. I have to stay here and work to pay off my rent, and then I’ll probably not have much time for practicing because I’m working, and then I’ll probably feel like I have not improved all summer at the end of it, and then again, I’ll be surviving and not thriving.*

*Group B Speaker 10*

Participants were left with insufficient funds to pay for health care when needed, either for maintaining wellness (gym membership, exercise classes, massage) or for therapeutic support not covered by the institute’s subsidy. The tension between needing and wanting to practice and struggling to make ends meet was described as exhausting and one of the main threats to psychological and physical health. Recommendations to address financial barriers are listed in Table 3.

**Table 3:** Student recommendations for change addressing “Finance” barrier.

1	Increase the stipend to match the rising cost of living
2	Either remove or significantly raise the cap on independent earnings
3	Review playing schedule to enable openings for paid work
4	Determine a health budget for each student that can be used according to need, for example funds currently assigned for counselling could be used for physiotherapy instead
5	Set up a small gym where students can put into practice physiotherapy recommendations for playing fitness
6	Morning yoga classes on site

#### Leave

Participants described having insufficient time off to have a healthy private and social life alongside the institute’s schedule. They spoke of the difficulty of having leave approved even when the schedule was quiet, making it hard to organize paid work, including opportunities for external orchestral work with a potential impact on future employment.

*I think there could be more allowance with leave requests… I’ve just heard of lots of people getting denied leave which the instances seem like they should have definitely been approved for leave and then they end up having to do this concert that they barely played in, or they could have easily been replaced for and it definitely seems like that’s a big stressor for a lot of people. Especially when the leave is to do something like a professional gig where what we are here to do is prepare for those, you know, to be professional.*

*Group B Speaker 3*

While sometimes students were given several days off at a time, at other times the rehearsal and performance schedule meant they might be required for 2 or 3 weeks straight without a break. Travel to distant venues reduced potential time off. Participants experienced snowballing fatigue because of having to play catchup during scheduled breaks. Scheduling without the opportunity for a reasonable break was seen as unsustainable, incompatible with self-care, and a source of distress.

*In semester 1 my friend did not have a single Saturday off. That was 13 Saturdays in a row and there were going to be 4 weeks where she did not have a Saturday or a Sunday off. I think that is due to the nature of the instrument she plays, in that that instrument family plays with the strings and the winds and brass in everything. But I just think if you want us to be well, do not make someone work 13 Saturdays in a row.*

*Group D Speaker 11*

*And fatigue really snowballs as well. Like being busy for one period and then something else comes up, and something else keeps coming up… All of a sudden we had an empty week, we can rest there, all of a sudden, [it’s] just as busy as the last one.*

*Group C Speaker 3*

Participants recommendations for addressing the “Leave” barrier are listed in Table 4.

**Table 4:** Student recommendations for change addressing “Leave” barrier.

1	Structure timetable for at least 1 day off a week. If there have been rehearsals/ concerts over the weekend, then schedule Monday or Tuesday off
2	Consider approving leave if there is no or minimal playing requirement at the institute at that time
3	Never schedule institute activities on a public holiday

#### Time

While they felt well-informed about general mental and physical self-care, participants said that lack of time restricted their resources and opportunities for effective self-care. Examples offered included insufficient time to engage in regular exercise, buy and prepare healthy food, get adequate sleep or rest, visit health care practitioners, or see family and friends. They experienced this situation as unsustainable, with some participants showing signs of distress during the discussion.

*I would say the no days off… because from a physical health point of view it’s unsustainable. But also from a mental and social perspective just actually having time to see your family and see friends and actually be able to socially engage, because by the time you are finished with your studies, then you go and work your part time job because your financial assistance is not enough to live off, there is not many hours left in the day.*

*Group A Speaker 4*

*What I would say is I think that by this point we are all very educated on what, in an ideal world, we would need to do. We’re all aware of how to eat properly, how much exercise, and what sort of exercise we need to do. We’re well aware of the treatment options that are available should we get injured, etc. But as we said, if you just do not have the time to do it and get everything done, you are not going to do it.*

*Group A Speaker 2*

*I think most of us can be aware that we are working too hard or doing something’s not good for our well-being, but there’s still that class tomorrow that you have to practice for and you are going to pick your instrument over your physical and mental health every time… I think you can sometimes feel like you are kind of fighting the schedule or something to find time for yourself. Say if you have got a busy week and you barely have any time to think, let alone check in with yourself. Which is not necessarily a problem for a short amount of time, but if it grows and grows and 1 week turns into a couple months, then it can become a serious issue.*

*Group B Speaker 3*

Time pressures were also seen as causing people to miss performance classes, which impacted a sense of cohesion among the student cohort. Although the institute scheduled some social activities, participants spoke of insufficient time spent together to bond as a whole group, with a tendency for instrument groups to remain siloed.

*What I felt I’ve missed is organized social activities… In a way I feel like there can be activities designed to sort of get people talking to people they might not normally. I feel like there’s a very big divide between the strings and everyone else.*

*Group C Speaker 4*

Participant recommendations to address time constraints are listed in Table 5.

**Table 5:** Student recommendations for change addressing “Time” barrier.

1	Structure the timetable for at least 1 day off each week
2	Increase stipend to reduce the need for outside earnings
3	Teachers plan rosters in dialogue with students; perhaps a section meeting at the beginning of semester where the teacher explains the roster and invites input
4	Possible exchanges between students on the playing roster
5	Encourage class attendance as a performance opportunity in front of peers
6	More regular social events along the lines of Baroque dancing, staff concerts, gatherings around food and playing sport

#### Pressure

Participants experienced pressure from multiple sources: having insufficient downtime; successive unrelenting deadlines; pressure from wanting to keep up with peers, pressure from one’s own self-expectations that result in unhealthy behaviors; lack of control over one’s own time and financial status; difficulty of scheduling timely lessons with peripatetic staffing, and students being responsible for staying within the lesson budget.

*I think within music… there’s a degree of unspoken competition between the players. This is not a spoken thing, but you definitely do not want to be the worst [musician] there. And so a lot of motivation, especially for me, is just trying to keep up. And I think that’s a limiter because in regards to especially mental health… It just kind of weighs on my mind. I think that’s a bit of a limiter because even though it’s such a positive environment, such a great place to study, and everybody here is really positive and a great influence - it’s just still the industry itself is competitive I think. Or maybe I’m just a competitive person, but - it’s just hard to kind of mitigate that in one’s mind.*

*Group B Speaker 1*

*For me whenever I practice, there’s some sort of degree of almost cognitive dissonance or something if I feel in my mind that I have not done enough for today… I always set this goal of 3 h a day and I have a lot of commitments outside of music that it’s almost impossible sometimes to put that in the day within reason. And so if I even go to the degree of doing 2 h and 40 min, it weighs on my mind for the rest of the day, and oh I need to do 20 min or I have not done my work. And I might have done, I might have maxed out my work, I might have done lots of really great work on something and I was like, I know it’s plenty of work done for today, but it just does not sit right in my mind.*

*Group A Speaker 6*

Some participants raised the uncertainty of career prospects and their futures generally, and a few experienced pressure from unprofessional behavior onsite, including bullying.

*I struggle with the uncertainty of not knowing where you might get work in the future, what country it might be in, how far out of your comfort zone you’ll have to go. To perhaps find the smallest amount of work. Yeah. Struggle with that and the future being so unknown.*

*Group A Speaker 8*

Participant recommendations to address issues around pressure are listed in Table 6.

**Table 6:** Student recommendations for change addressing “Pressure” barrier.

1	Schedule fewer than 9 h of performance classes in a week
2	Use performance classes to lead into a social opportunity
3	Schedule performance classes in the afternoon for more reliable attendance
4	Education sessions on emotional intelligence, ethical behavior, professionalism, and culture/behavior expected at the institute
5	Skills training in responding to inappropriate behavior in others, including bullying
6	Training in self-reflection and self-awareness (this already happens in various contexts across the faculty in relation to performance)
7	Manage student wellbeing on an individual level, similar to elite sport

#### Program structure and content

Further comments regarding the structure and content of the wellbeing program included:

- Some activities and presentations were insufficiently targeted to musicians. Participants noted that lectures or activities needed to show a practical application to music performance and to them as musicians to be seen as relevant.
- Practical and hands-on classes were more readily integrated than lectures, while large amounts of information and presentations not systematically structured or connected could be repetitive or disjointed, and difficult to integrate and put into practice.
- Wellbeing sessions scheduled for the morning or on days that were already heavily scheduled increased the pressure burden, creating resentment rather than achieving their purpose of supporting wellbeing or relieving stress and symptoms. The sense of resentment particularly applied to sessions that were over 45 min in length which started to encroach on practice time.

Student recommendations to improve the wellbeing program structure and content are listed in Table 7.

**Table 7:** Student recommendations for change addressing “Program Structure and Content” barrier.

1	More sessions like those scheduled with high-level musicians during lockdown, where those musicians spoke about their journeys and careers
2	More experiential/ hands-on classes
3	Create a framework for wellbeing information so it is organized, accessible, relevant, avoids unnecessary repetition, and can readily be put into practice
4	Have wellbeing practitioners attend more than once to develop information that has been offered previously
5	Do not schedule wellbeing sessions in project weeks
6	Schedule wellbeing sessions at the end of the day with the possibility of everyone gathering afterwards for food/ drink

### Prioritized barriers and recommendations

Forty-nine students (response rate 74%) ranked their top five points on each of the two documents *Barriers to Wellbeing* and *Recommendations for Change* (Supplementary materials 2, 3) in order of importance from 1 to 5. Table 8 shows the five barriers to wellbeing nominated most frequently, with the percentage of students who placed each one in the top five. Table 9 shows the five recommendations for change nominated most frequently, with the percentage of students who placed each one in the top five.

**Table 8:** Barriers to wellbeing ranked by participants in the top 5.

Barrier category	Specific problem	% of students who placed problem in top 5
Financial	Stipend insufficient to cover basic expenses	72%
Leave	The difficulty of having leave approved to engage in outside work, including opportunities for orchestral work	60%
Time	Conflict between playing demands and time for mental/ physical/ social self-care	56%
Financial	Low cap on allowable earnings	44%
Time	Unsustainable weekly playing schedule when there are no days off	38%

**Table 9:** Recommendations for wellbeing ranked by participants in the top 5.

Recommendation category	Specific recommendation	% of students who placed recommendation in top 5
Financial	Update policy around stipend (e.g., allow higher earnings from outside work/abolish earnings cap)	46%
Leave	Consider approving leave if there is no or minimal playing requirement at the institute over the time requested	44%
Leave	Modify playing schedule to ensure at least 1 day off each week	36%
Health and fitness	Set up a small gym where students can put into practice physiotherapy recommendations for playing fitness	36%
Social	Regular “Circuit-breaker activities” for social bonding and musician enhancement, e.g., staff concerts, Baroque dancing, pizza afternoons	30%

## Discussion

This study identified systemic practices around finances, time, leave, and pressure as main themes affecting student wellness at an elite pre-professional music training institute. The focus group discussions from which these themes were drawn centered around cultural and institutional attitudes and policies, which reflected the multilayered frameworks that formed the theoretical background for this study (Healthy Conservatoires, 2020; Michie et al., 2011; NIOSH, 2016; WHO, 1986). In contrast to the more usual situation of wellbeing initiatives in music faculties either being optional or not offered at all (Wijsman and Ackermann, 2019), an unusual feature of this institute is that all students are expected to engage with its comprehensive wellbeing program. A similar study conducted elsewhere may have yielded results that focused on the need for wellbeing offerings to be made available, but as these were already in place, this study moves to the foreground the crucial role of institutional policies and cultural mores in musicians’ health.

Previous studies have discussed the need to address the conservatoire culture and its structures and processes (Araújo and Spahn, 2022; Araújo et al., 2017; Matei and Ginsborg, 2022; Perkins et al., 2017), however, this is the first study to our knowledge to formulate a design process for a musicians’ health protocol using recommendations from occupational and public health as well as behavior change perspectives. Where efforts in improving the health status of musicians have historically been focused on conveying knowledge and skills, with more attention over the past 5 years given to behavior change (Matei and Ginsborg, 2022; Norton, 2020), this study shifts that focus to addressing organizational systems and attitudes as a priority as emphasized in occupational health (NIOSH, 2016).

### The institute

The institute where this study was conducted is committed to equipping its students for successful music performance careers. This has included dedicating significant resources to student health and wellbeing. Philanthropic funding and the administration’s attention to wellbeing means that it is arguably better-resourced than most music faculties and able to provide a content-rich wellbeing program, including many of the aspects proposed in Matei and Phillips (2023) (see also Rosset et al., 2022). At the time of the study, the institute’s wellness protocol addressed all eight domains of the Healthy Conservatoires UK Fit to Perform framework (Healthy Conservatoires, 2020), in contrast to the widespread lack of health knowledge and skills available at most tertiary music faculties (Wijsman and Ackermann, 2019).

While participants expressed appreciation and gratitude for the wellbeing program and for the opportunity to study at the institute itself, they noted the dislocation between what they had learned and been advised to do, and the absence of time, opportunity and energy to do it due to financial and time constraints and practice and performance demands. This dislocation between stated policy and achievable practicality has been previously signposted by Matei and Ginsborg (2022) and Araújo and Spahn (2022). The situation where participants felt they were not thriving but struggling to survive (Keyes, 2009) illustrates the application of NIOSH (2016) hierarchy of control and Michie et al.’s (2011) COM-B model, suggesting barriers to health at the level of organizational policy and culture can make it difficult if not impossible for individuals to practice effective health literacy.

Music students have been reported as exercising low responsibility for their own health (Araújo et al., 2017), however, perhaps part of the reason they do not exercise adequate self-care is because of the logistic and cultural environments in which they operate. The established approach of directing health interventions toward the behavior of individual musicians without addressing the overarching policies and the cultural environment could be an explanation of why the prevalence of playing-related problems has remained persistently high. However, it remains to be seen whether the health of musicians improves in healthier music study and work environments. It seems easier to place full responsibility for exercising health literacy on musicians by providing them with information and strategies, than to face the formidable challenges of institutional and cultural change. However, the interdisciplinary literature suggests that unless those needed changes are addressed, strategies aimed at increasing the knowledge and skills of individuals are unlikely to bring about sustained improvements in wellbeing (Kelly and Barker, 2016; Lowe, 2004; NIOSH, 2016; Nutbeam, 2019; Weale et al., 2022).

### Barriers to wellbeing and recommendations for change

Among the four main themes of finances, leave, time and pressure derived from the focus group data, barriers most often prioritized in the student ranking fell into the finances, time and leave themes, whereas the most frequently prioritized recommendations for change included fitness and exercise and social connection. This flags the importance of returning to stakeholders for consideration of issues that may initially have been nominated by a minority in one format, as these issues may come to the fore when presented back in another format.

Three of the four main themes: finances, time and leave, were all discussed by participants in terms of instability and insufficiency. Conflict between financial and time constraints and practice and performance demands compromised participants’ self-care even though they were well-informed and possessed a good understanding of wellbeing. Participants did not regard the problems as unsolvable, putting forward clear recommendations to allow more flexibility in the schedule (See Tables 3–5 and Supplementary material 3 for the full list of recommendations). However, the strain around finances leading to problems with mental health echoed Gross and Musgrave’s (2016) findings that the leading cause of mental health difficulties in professional musicians in the UK was financial and vocational precarity.

A willingness to negotiate flexible solutions was more prominent in focus group discussions than were requests for more services or less work, reflecting participants’ interest in constructive engagement with problem-solving when it came to barriers to wellness, and their desire for more autonomy. For example, when discussing factors contributing to pressure, participants spoke of sometimes being required for 2 or 3 weeks’ performing without a day off, during which time other aspects of life banked up. When days off did arrive, participants used them to try to catch up on earnings and other commitments, causing snowballing fatigue. To help counteract these unsustainable conditions, participants requested more input into rehearsal and performance scheduling, introducing the possibility of being able to swap with someone else if logistics allowed it. The request for active participation in scheduling is consistent with the appreciation participants had for everything the institute had to offer while increasing their autonomy within it. Autonomy is named as one of three core psychological aspects of wellness in Self-Determination Theory (Evans, 2015).

One way of analyzing focus group discussion data is noting how long the group spends on a topic or how many people speak on it. However, that has the potential to underplay the importance of topics that might be more difficult to bring into a group discussion. This includes issues that came under the theme of internal pressures. Isolation from family and friends, and limited opportunities to socialize with fellow students due to packed schedules compromised relatedness, which is one of Self-Determination Theory’s three essential components for wellbeing (Evans, 2015). While many participants were happy with the support structures the institute provided, several had experienced repeated difficulties with feeling unheard and felt they were regarded as the problem, instead of the problem being the one they were trying to bring forward. Similarly, there was some discussion around unprofessional behavior and bullying that gave rise to recommendations for training in ethics and emotional intelligence. Though the voices were few on these matters, there was intense silence through these parts of the discussion, which raises the question of how many others felt the same but were reticent to speak about it. Bullying and micro aggression can potentially play into the perfectionism prevalent in this population and undermine a sense of competence, which is another component of wellbeing.

Several participant barriers and recommendations were similar to those put forward in Matei and Ginsborg’s (2022) analysis of a 5-month wellbeing course with UK conservatoire students, for example, participants felt that lack of specificity to their situation and too much overlap in the material was a waste of their time; they wanted more sessions with high-level musicians telling their stories and offering guidance, and they wanted more “hands-on” practical sessions that applied directly to playing their instrument. In addition, participants in this study reflected that health protocol content would be easier to absorb if it was more structured and the topics progressed logically.

This study supports public and occupational health statements that even in the presence of a comprehensive, well-resourced wellbeing program, playing-related problems arise in the context of personal, institutional, and social practices and attitudes that can influence health behavior more strongly than health literacy (Nutbeam, 2019), and that psychosocial as well as other health determinants need to be addressed at the organizational level (CSA Group and BNQ 2013/2018; Michie et al., 2011; Oakman et al., 2018). The inclusion of cultural and organizational factors in participants’ perception of factors contributing to playing-related problems shows the utility of the multiple levels in the COM-B behavior model when considering prevention and management of playing-related problems, and illustrates the congruence between the COM-B model and occupational and public health frameworks for use in the tertiary music context.

### Author recommendations for revised health protocol

At the time the overall project ended at the end of Step 4 of the 7-step process, and subject to potential adjustments in response to further data collected in Steps 5–7, the working plan was to apply the following principles, design and content delivery to the existing protocol:

#### Principles

1. Engaging curricula and learning experiences.
2. Supportive social, physical and digital information exchange environments.
3. Community awareness and actions involving all levels of the organization.
4. Development of students’ mental health knowledge and self-regulatory skills.
5. Access to effective services.
6. Ongoing evaluation at institutional and individual levels, with responsive policy and program adjustment.

#### Design

Provision of early detection and intervention for mental and physical health symptoms progressing through the following four levels with levels 1 and 2 addressed in the education program:

1. Preventative or ‘foundational’ components;
2. Indicated (at-risk) prevention components;
3. Early intervention;
4. Access to musician-specific health care.

#### Education program content delivery

*Modularized*: Modules to cover each element of the Healthy Conservatoires 8-factor wellbeing framework (Figure 1).

*Scaffolded*: To clarify content structure across the year-long program, each module begins with an introductory session, for example for a mental health module, Emotional WB 1.0 Introduction, which expands and develops in complexity in subsequent modules, for example Emotional WB 2.0 Mindfulness; 2.1 Mindfulness for Optimal Performance; Emotional WB 3.0. Performance anxiety; 3.1 Arousal regulation; 3.2 Self-talk; 3.3 Performance routines; Emotional WB 4.0 Mood management etc.

*Scheduled*: One module delivered in at least one session per week during term time.

*Measured*: Students complete validated outcome measures of mental and physical health and wellbeing on commencement of the program and after each term to track progress, flag areas of risk and those needing remediation.

*Applied:* Students complete short learning summaries at the end of each session and set wellbeing action points for how they intend to apply that learning over the coming week into their practice sessions, rehearsals, performances, and/or their lives outside of music. Instrumental teaching faculty are aware of the worksheets and ask students to review their intentions for healthy playing at the start of each lesson and rehearsal session.

### Future directions

It would be useful for future research to expand its focus from wellbeing strategies for individuals, to consider systemic influences at organizational and cultural levels as suggested by the interdisciplinary literature and by the results of this study. The music industry has a long history with established traditions and priorities that can make it challenging to integrate wellbeing as a key factor in music performance. However, traditions and priorities can be examined and tested over time to establish which must be constructively managed and which can be modified or discarded to achieve enhanced wellbeing as well as improved performance.

To move in the direction of cultural change, this study supports the use of multidimensional theoretical frameworks for designing and testing health initiatives for musicians, for example the Healthy Conservatoires Wellbeing Framework (2020) the COM-B behavior change model (Michie et al., 2011), and occupational and public health principles (CSA Group and BNQ, 2013/2018; Nutbeam, 2019; WHO, 1986).

### Strengths and limitations

The strengths of this study were that it was theoretically grounded across an array of disciplines; the two researchers are qualified in several music, health and education-related areas between them and thus brought a broad range of views and expertise to the project; participation rate in the focus groups was high; and unusually, the study was conducted with a multidisciplinary health protocol already in place at the institute. Synthesizing focus group results and returning them to students for rating in order of importance elicited a good response rate and provided triangulation of results, helping to offset researcher bias. Additional rigor was added to the study by the researchers working together in real time throughout project planning and data analysis, reflecting on assumptions we had made from our professional backgrounds, and contrasting study results with those we had expected.

Limitations of this study include that it is based on a single institution so the results are not necessarily generalizable, and the study was conducted by researchers with experience as healthcare practitioners and educators, but with no experience of working as professional musicians or music administrators. In addition, asking participants to prioritize their top five barriers to wellbeing and recommendations for change had the unintended effect of screening out potentially equally important barriers and recommendations suggested by a minority. These should be held over for reconsideration during future program reviews.

## Conclusion

Barriers to wellbeing in the context of elite pre-professional music training, and proposed recommendations for change to improve student wellbeing related mostly to institutional policy and culture. Psychosocial, organizational and cultural factors contribute to psychological and physical playing-related problems, and need to be addressed using a structured, integrated approach, with risk control implemented at the highest possible organizational level. Successful health promotion programs need to be theoretically sound and contextually appropriate; operate across every level of the institution; possess the committed support of managers and teachers; involve ongoing collaboration with all stakeholder representatives; build healthy environments; strengthen community action around wellbeing; improve individual health literacy, and prove to be effective in achieving specified wellness outcomes. Evaluation of the effect of wellbeing programs on the health of musicians and program adjustment in response to continual feedback and changing conditions is key to success.

## Data Availability

The datasets presented in this article are not readily available because ethics approval for this project does not permit sharing the collected data. Enquiries regarding the datasets should be directed to mosborne@unimelb.edu.au.
