# Supplementary material for: Wellbeing for young elite musicians: development of a health protocol from a student perspective
Source: Front Psychol. 2025 Feb 12;16:1401511. doi: 10.3389/fpsyg.2025.1401511 (PMC11861350; doi:10.3389/fpsyg.2025.1401511)
Supplement: Supplementary file 1 [file Data_Sheet_1.docx]

Supplementary Material 1: Focus group questionnaire

Wellbeing for young elite musicians: development of a health protocol from a student perspective

Ann Shoebridge, Margaret S. Osborne

*** Correspondence:** Margaret Osborne: mosborne@unimelb.edu.au

# Focus group discussion introduction and questions

**OUR BRIEF**

*What conditions and support services are needed for <name of institute> musicians to be mentally and physically safe and to thrive?*

*Today we’re here to learn how you perceive health and wellbeing at <name of institute> and hear your thoughts about how <name of institute> can best support this. We’ll start with a general question about musicians’ health and wellbeing, move on to your personal views and experiences, and finish with a discussion of what happens at <name of institute>, and possibilities for useful change.*

**GENERAL**

1. What comes into your mind when you think about musicians’ health and wellbeing?

**PERSONAL**

**2.** What is your attitude to health and wellbeing as a musician? Where does it sit with you?

***Probe*** *– does it change according to the circumstances or context you’re in?*

3. How is health and wellbeing expressed in your day-to-day practices? (what practical difference does it make?)

***If time* -** Who would you approach for advice/support/help with any health and wellbeing issues that may occur?

****AT 15 minutes****

*What I’d like you to think about now is what contributes to good health and wellbeing in musicians at your level of professional development; what contributes to an experience of ill-health, and how <name of institute> might best work in this area.*

**ACADEMY**

4. What have you experienced since studying at *<name of institute>*, if anything, that you feel has supported or enhanced your health and wellbeing?

***Probe****: Details, experiences, why it was supportive, who enabled it?*

A:  What aspects would you like retained? Why?

B:  How can the existing program be strengthened?

 5. Have you experienced or perceived any barriers to your health and wellbeing since being a *<name of institute>* student? Do you have any ideas of how such barriers might be dealt with?

***Probe****: Details, experiences, why it was a barrier?*

6. If there’s one thing you never wanted to see again, to chuck out, what would it be?

7. What would you like to experience at *<name of institute>*, in an ideal world, to support and enhance your health and wellbeing?

**Follow-up**

Is there anything else you would like to add to what we have discussed today?

**CLOSE**
