# Supplementary material for: Wellbeing for young elite musicians: development of a health protocol from a student perspective
Source: Front Psychol. 2025 Feb 12;16:1401511. doi: 10.3389/fpsyg.2025.1401511 (PMC11861350; doi:10.3389/fpsyg.2025.1401511)
Supplement: Supplementary file 2 [file Data_Sheet_2.docx]

Supplementary Material 2: Barriers to wellbeing

Wellbeing for young elite musicians: development of a health protocol from a student perspective

Ann Shoebridge, Margaret S. Osborne

*** Correspondence:** Margaret Osborne: mosborne@unimelb.edu.au

# Possible Barriers to Wellbeing distributed for ranking

| **Topic categories** |  | **Rank the top five items that most strongly impact your wellbeing** |
| --- | --- | --- |
| 1. *Time limitations* | Unsustainable weekly playing schedule when there are no days off |  |
|  | Insufficient openings in the schedule to earn a living |  |
|  | Conflict between playing demands and time for mental/ physical/ social self-care |  |
| 1. *Financial challenges* | Stipend insufficient to cover basic expenses |  |
|  | Low cap on allowable earnings |  |
|  | Reported earnings covers only one month instead of also covering non-earning months |  |
|  | Cost of health care makes it difficult to access |  |
|  | People who handle heavy and awkward instruments and who are under pressure can injure themselves, but have no financial cover for treatment |  |
|  | Student services fee |  |
| 1. *Decreasing number of teaching hours and financial assistance with each year* |  |  |
| 1. *Lack of control over your own time and financial status* |  |  |
| 1. *Leave* | The difficulty of having leave approved to engage in outside work, including opportunities for orchestral work |  |
| 1. *Social environment* | 1. Lack of time or space to bond as a whole group |  |
|  | 1. Heavy work schedule precluding time with family or with other academy people |  |
| 1. *Pressure to conform to unhealthy behaviours* | From teachers |  |
|  | From peers |  |
|  | From yourself |  |
| 1. *Unprofessional behaviour* |  |  |
| 1. *Bullying* |  |  |
| 1. *Response to complaints* | 1. Issues raised with staff do not generate satisfactory outcomes |  |
|  | 1. Issues raised with staff are met with responses about policy/ procedures that are not understandable |  |
|  | 1. Students raising an issue can feel like they are the problem |  |
|  | 1. Being afraid to raise an issue because of the potential impact on your career |  |
| 1. *Other (Please describe)* |  |  |
