# Supplementary material for: Wellbeing for young elite musicians: development of a health protocol from a student perspective
Source: Front Psychol. 2025 Feb 12;16:1401511. doi: 10.3389/fpsyg.2025.1401511 (PMC11861350; doi:10.3389/fpsyg.2025.1401511)
Supplement: Supplementary file 3 [file Data_Sheet_3.docx]

Supplementary Material 3: Recommendations for Change

Wellbeing for young elite musicians: development of a health protocol from a student perspective

Ann Shoebridge, Margaret S. Osborne

*** Correspondence:** Margaret Osborne: mosborne@unimelb.edu.au

# Recommendations for Change distributed for ranking

| **Issue** | **Rank the top five items that you wish to see implemented** |
| --- | --- |
| Modify playing schedule to ensure at least 1 day off each week |  |
| Create a playing schedule that makes it possible to balance playing demands and self-care |  |
| Teachers plan rosters in dialogue with students |  |
| Schedule fewer than nine hours of performance classes a week |  |
| Use performance classes to lead into a social opportunity |  |
| Schedule performance classes in the afternoon for more reliable attendance |  |
| Provide consistent lesson times and income across instrument groups and years of study |  |
| Regular (weekly?) “Circuit-breaker activities” for social bonding and musician enhancement, e.g. staff concerts, Baroque dancing, pizza afternoons |  |
| No academy activities scheduled on public holidays |  |
| Update policy around stipend (increasing stipend amount, allowing higher earnings from outside work) |  |
| Allow time in the playing schedule to work for income |  |
| Consider approving leave if there is no or minimal playing requirement at the academy over the time requested |  |
| Address pervasive cultural norms around quantity of practice and not taking sufficient breaks |  |
| Provide education around respectful and professional behaviour |  |
| Skills training in responding to inappropriate behaviour in others, including bullying |  |
| Training in self-reflection, self-awareness |  |
| Establish a clear, accepted complaint and response process that reliably delivers transparent positive outcomes |  |
| Staff awareness and understanding of individual student health needs |  |
| Have a health budget for each student that can be used according to need e.g. funds currently earmarked for a psychologist could instead be used for physiotherapy. |  |
| Create a framework to scaffold wellbeing information so it is organised, accessible, relevant, avoids unnecessary repetition, and can readily be put into practice |  |
| No wellbeing sessions scheduled during project weeks |  |
| Schedule wellbeing sessions at the end of the day with the possibility of everyone gathering afterwards for food/ drink |  |
| More sessions like those scheduled with high-level musicians during lockdown, where they speak about their journeys and careers |  |
| More experiential/ hands-on classes |  |
| Set up a small gym where students can put into practice physiotherapy recommendations for playing fitness |  |
| Morning yoga classes |  |
| Manage student wellbeing on an individual level |  |
| Abolish student services fee |  |
| Other (Please describe) |  |
