# Supplementary material for: Wellbeing for young elite musicians: development of a health protocol from a student perspective
Source: Front Psychol. 2025 Feb 12;16:1401511. doi: 10.3389/fpsyg.2025.1401511 (PMC11861350; doi:10.3389/fpsyg.2025.1401511)
Supplement: Supplementary file 4 [file Data_Sheet_4.docx]

Supplementary Material 4: Pre-intervention wellbeing offerings

Wellbeing for young elite musicians: development of a health protocol from a student perspective

Ann Shoebridge, Margaret S. Osborne

*** Correspondence:** Margaret Osborne: mosborne@unimelb.edu.au

# Pre-intervention wellbeing offerings at an elite pre-professional music academy

| **Wellbeing topic** | **Specific offerings** |
| --- | --- |
| Musculoskeletal | Self-care  Injury prevention  Recovery from fatigue  Recovery from injury  Applied physiology  Breathing for wind and brass  Individual physiotherapy consultation  Chronic pain  Sleep for optimal performance  Nutrition  Audiology lecture and assessment |
| Psychological | The academy is a safe place  High-pressure environments – what to expect  Mindfulness  Music performance anxiety  Perfectionism  Impostor syndrome  Self-identity  Values  Self-esteem  Self-compassion  Resilience – dealing with setbacks  Confidence  Emotional regulation  Social anxiety  Drugs & alcohol |
| Psychophysical | Breathing and bodywork using yoga  Relaxation  Guided meditation  Beta-blockers |
| Social/ Spiritual/Environmental | Indigenous cultural awareness & environment  Ecopsychology – immersion in nature  Music and the brain  Neurodiversity  Gender identity  Beliefs, values and ethics  Creating purpose and meaning  Navigating social media  Public media training |
| Career skills | Aspirations and goals  Motivation  Stress  Workload management  Performance scheduling  Job opportunities  Managing contracts  Career transition  Managing money  Grant-writing  Global environment  Life on trial  Creative curiosity & lifelong learning |
